# Treatment of vascular dementia based on neuroprotection and neural repair strategies: from pathophysiology to natural products

**DOI:** 10.3389/fphar.2026.1942636

**Published:** 2026-10-20

**Authors:** Qinzhe Luo, Zhaoxing Li, Yuyuan Liao, Wenshuo Chen, Zhihong Xu, Xin You, Guannan Xue, Yan Duan, Juan Li, Shunxiang Li

**Affiliations:** 1 School of Pharmacy, Hunan University of Chinese Medicine, Changsha, China; 2 Hunan Engineering Technology Research Center for Bioactive Substance Discovery of Chinese Medicine, Hunan University of Chinese Medicine, Changsha, China; 3 Hunan Province Sino-US International Joint Research Center for Therapeutic Drugs of Senile Degenerative Diseases, Hunan University of Chinese Medicine, Changsha, China; 4 School of Pharmacy, China Pharmaceutical University, Nanjing, China

**Keywords:** natural products, vascular dementia, pathological mechanism, neuroprotection, neural repair

## Abstract

Vascular dementia (VaD) is a heterogeneous cognitive disorder associated with cerebrovascular pathology and persistent vascular dysfunction. Because effective disease-modifying therapies remain limited, natural products have attracted interest as potential interventions for the complex pathology of VaD. This review examines their effects from two complementary perspectives: neuroprotection and neural repair. Experimental studies indicate that natural products can influence several interconnected processes, including redox imbalance, neuroinflammation, cell survival, blood–brain barrier dysfunction, vascular remodeling, neurogenesis, and synaptic plasticity. However, the available evidence varies considerably among isolated compounds, botanical extracts, and herbal formulations. Some isolated compounds have been supported by relatively coherent relationships between molecular targets, tissue responses, and functional outcomes, whereas evidence from clinical studies remains much more limited. We therefore consider the current mechanistic evidence together with the major challenges in pharmacokinetics, quality control, and clinical translation.

## Graphical Abstract

**Figure:**
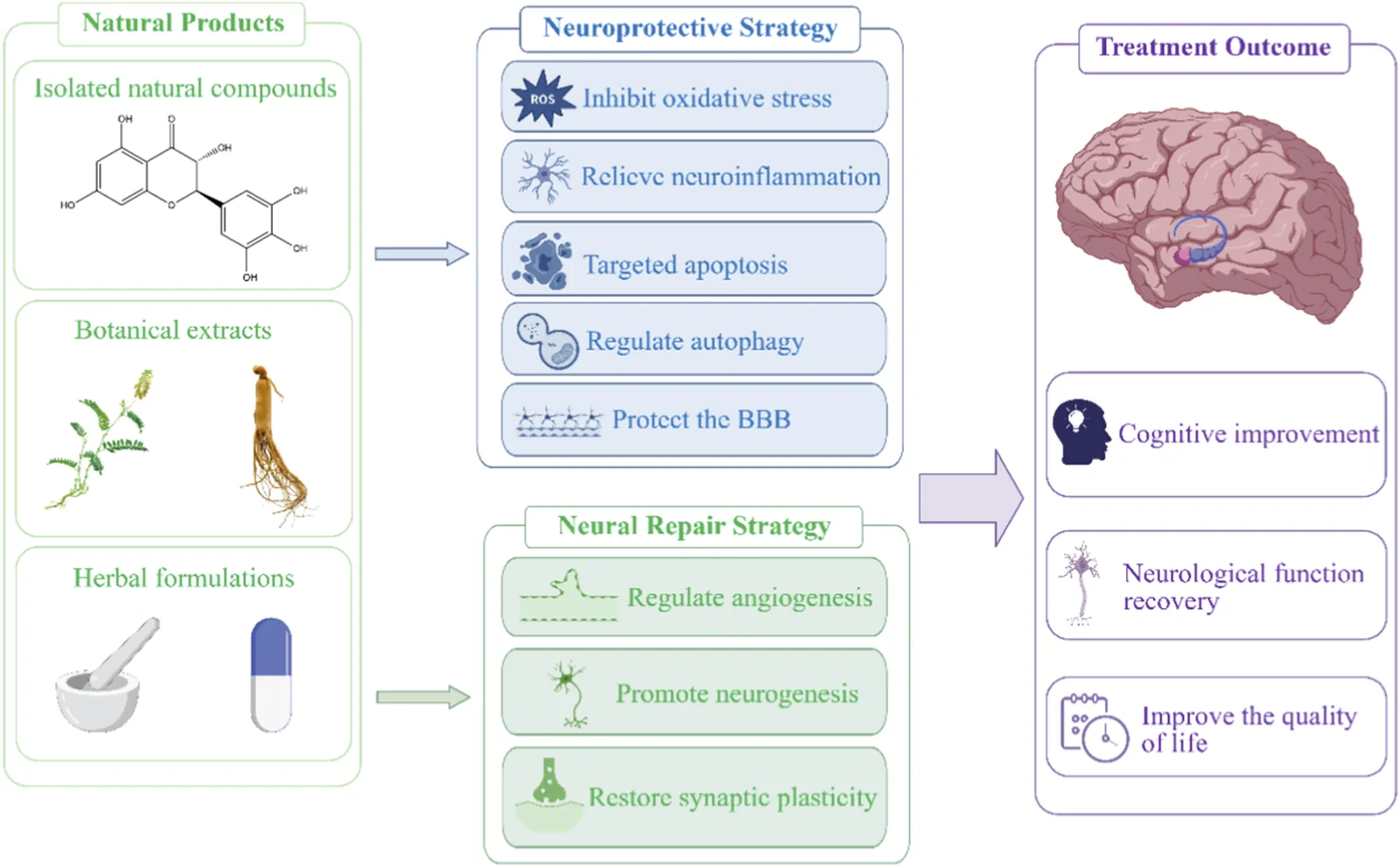
Created with BioGDP.com (Jiang et al., 2025).

## 1 Introduction

Vascular dementia (VaD) is a heterogeneous group of disorders in which cerebrovascular disease and the resulting brain injury lead to progressive cognitive decline (O’Brien and Thomas, 2015). It is the second leading cause of dementia after Alzheimer’s disease and has become an important global health concern. In 2021, an estimated 56.9 million people were living with dementia worldwide, and this number is projected to reach approximately 137.6 million by 2050. If current epidemiological proportions remain broadly unchanged, the number of people affected by pure VaD and mixed vascular–degenerative dementia could reach about 42.7 million by 2050 (Cai et al., 2026). The economic consequences are also substantial. Estimates based on the value of statistical life approach suggest that the global economic burden of Alzheimer’s disease and related dementias will rise from approximately $2.8 trillion in $2019 to $16.9 trillion by 2050, with an increasing share of this burden borne by low- and middle-income countries (Nandi et al., 2022). These trends underscore the need for effective interventions targeting the complex pathology of VaD.

The pathological progression of VaD is dynamic and continuous. Cerebrovascular injury and persistent cerebral hypoperfusion may accumulate over time, leading to dysfunction of neurons, white matter, and neural networks and ultimately manifesting as cognitive decline. During this process, oxidative stress, neuroinflammation, neuronal apoptosis, autophagy dysregulation, blood–brain barrier (BBB) disruption, and abnormalities in neurovascular coupling and synaptic function interact with one another and contribute to sustained pathological changes (Kuang et al., 2021). The duration and relative contribution of these processes may vary throughout disease progression. VaD is therefore better understood as a continuous pathological process extending from vascular injury to neuronal, white matter, and neural network dysfunction rather than as a static and homogeneous disease entity.

This pathological complexity indicates that treatment of VaD should address not only ongoing and secondary brain injury but also the recovery of damaged neural tissue and network function. Current clinical management mainly includes vascular risk-factor control, secondary stroke prevention, lifestyle modification, and symptomatic treatment, whereas disease-modifying therapies directed at the core pathology of VaD remain limited. Drawing on therapeutic concepts established in ischemic brain injury, neuroprotection and neural repair can be considered complementary therapeutic dimensions (Francardo et al., 2017; Liu and Chopp, 2016). Neuroprotection primarily limits secondary injury and preserves the integrity of surviving cells and tissues, whereas neural repair supports restoration of damaged brain tissue and neural network function through vascular remodeling, endogenous neurogenesis, and synaptic and neural network remodeling (Venkat et al., 2018; Yu et al., 2023). These processes are not strictly discrete stages but interact and partially overlap throughout disease progression.

Against this background, natural products have attracted increasing attention. Isolated natural compounds, botanical extracts, and herbal formulations may act on multiple components of the pathological network underlying VaD, providing a potential means of modulating complex vascular–neural–immune interactions. However, multitarget activity does not necessarily translate into clinical benefit. Natural products may face limited oral bioavailability, restricted BBB permeability, variability in metabolic and structural stability, difficulties in quality standardization, and incompletely characterized drug interactions and safety profiles. Natural products with anticoagulant or antiplatelet activity may also increase bleeding risk when combined with conventional antithrombotic therapies (Liu et al., 2018). Thus, their multicomponent and multitarget properties may offer a potential systems-level therapeutic advantage while simultaneously increasing the complexity of mechanistic interpretation, quality control, and clinical translation.

This review distinguishes natural products into isolated natural compounds, botanical extracts, and herbal formulations and evaluates their effects on VaD in relation to the underlying pathological processes and molecular and cellular mechanisms. Particular attention is given to the temporal interaction among pathological processes and to differences in evidence from cellular, animal, and clinical studies. Mechanistic associations are distinguished from experimentally supported target effects to avoid direct extrapolation of preclinical findings to patients. The aim is to define the major mechanisms of natural-product intervention in VaD while assessing the strength and limitations of the current evidence and the principal barriers to clinical translation.

## 2 Diagnosis, biomarkers, and classification of vascular cognitive impairment and dementia

VaD is associated with age, genetic susceptibility, and vascular risk factors including hypertension, diabetes, smoking, and cerebrovascular disease (Lecordier et al., 2021; Wolters and Ikram, 2019). The presence of vascular risk factors alone, however, does not establish a diagnosis of VaD. The updated VasCog-2-WSO criteria place vascular cognitive impairment and dementia (VCID) within a continuum that includes preclinical, mild, and major cognitive impairment, with VaD referring to the dementia-level phenotype in which vascular pathology makes a substantial contribution to cognitive decline. Diagnosis requires a combination of the clinical cognitive profile, evidence of cerebrovascular disease, and the temporal and pathological relationship between the two, together with consideration of alternative or coexisting causes. MRI remains an important tool for assessing cerebrovascular lesions, including lacunar infarcts, white matter hyperintensities, cortical infarcts, and cerebral microbleeds. Fluid biomarkers can provide complementary information: neurofilament light chain may reflect axonal injury, whereas cerebrospinal fluid or plasma amyloid-β and phosphorylated tau can help identify concomitant Alzheimer’s disease pathology (Sachdev et al., 2025). At present, these biomarkers are best regarded as supportive measures of vascular brain injury and coexisting neurodegenerative pathology rather than as stand-alone diagnostic markers of VaD.

Advances in multi-omics, liquid biopsy, and artificial intelligence have led to the identification of a growing range of candidate biomarkers for VCID, including C1q family proteins, melanoma inhibitory activity protein, CD163, placental growth factor, NfL, and glial fibrillary acidic protein (Che et al., 2025; Chen et al., 2025; Hosoki and Sachdev, 2024; Xu et al., 2025). These markers capture different components of the disease process, ranging from complement activation and immune-inflammatory responses to vascular dysfunction and neuronal or glial injury. Their potential clinical value is nevertheless still being established, as most have not undergone sufficient external validation and their performance may be affected by patient heterogeneity and the frequent coexistence of vascular and neurodegenerative pathology. A single biomarker is therefore unlikely to capture the full biological spectrum of VCID. A combination of clinical phenotyping, cerebrovascular imaging, and complementary biomarker profiles may provide a more informative basis for diagnosis and disease stratification.

From a classification perspective, the recently revised VasCog-2-WSO criteria place less emphasis on traditional subtypes such as post-stroke dementia, subcortical ischemic VaD, and multi-infarct dementia. Instead, greater emphasis is placed on the clinical severity of cognitive impairment and its underlying vascular etiology, with major categories distinguished according to the predominant vascular pathology, including ischemic, hemorrhagic, and mixed forms. In individuals with coexisting neurodegenerative pathologies, such as Alzheimer’s disease, the relative contributions of vascular and nonvascular pathologies to cognitive impairment should be further delineated (Sachdev et al., 2025).

Thus, the diagnostic focus of VaD and VCID has gradually shifted from simply determining whether a patient has VaD toward defining the underlying vascular etiology, cerebrovascular pathological burden, and coexisting disease processes. Accurate characterization of these pathological phenotypes is not only fundamental to the management of vascular risk factors and prevention of recurrent cerebrovascular events, but also provides a basis for subsequent neuroprotective and neural repair strategies aimed at limiting secondary brain injury and promoting the recovery of damaged brain tissue and neural networks.

## 3 Pathophysiology of vascular dementia

The pathophysiology of VaD is also heterogeneous. Different forms of cerebrovascular injury can produce sustained disturbances in cerebral perfusion and neurovascular unit function, ultimately affecting neurons, white matter, and synaptic networks (Dichgans and Leys, 2017; Jellinger, 2026; Morgan and Mc Auley, 2024; Ng et al., 2025). The underlying vascular pathology mainly involves cerebral small vessel disease and large vessel disease (Arvanitakis et al., 2016). Small vessel disease includes lacunar infarcts, microinfarcts, cerebral microbleeds, arteriolosclerosis, intracranial atherosclerosis, and cerebral amyloid angiopathy, and is associated with cerebral hypoperfusion, BBB dysfunction, impaired interstitial fluid and cerebrospinal fluid drainage, and vascular inflammation (Inoue et al., 2023). Large territorial infarction is a major consequence of large vessel disease and an important substrate of post-stroke dementia, although the clinical phenotype also depends on the extent of vascular injury and the presence of underlying neurodegenerative pathology (Iadecola et al., 2019). Atherosclerosis is another major contributor to large vessel disease and is closely linked to VaD (Gustavsson et al., 2020). As atherosclerotic plaques enlarge, progressive luminal narrowing can compromise cerebral blood flow and contribute to chronic cerebral hypoperfusion (CCH)(Huang Y. T. et al., 2021).

Persistent vascular dysfunction can then trigger a range of downstream cellular and molecular changes, including BBB disruption, neuroinflammatory activation, and loss of redox homeostasis, all of which may contribute to cognitive impairment (Yang, 2025). These processes are closely interconnected rather than occurring in isolation, and their interactions may sustain and amplify the pathological changes that ultimately underlie cognitive dysfunction. This continuum from vascular pathology to secondary brain injury provides the biological basis for neuroprotective and neural repair interventions in VaD (Figure 1). Neuroprotection primarily aims to limit secondary injury and preserve the structural and functional integrity of neurons and brain tissue, whereas neural repair extends beyond injury control to promote vascular remodeling, endogenous neurogenesis, and synaptic and neural network remodeling, thereby facilitating functional recovery of the injured brain.

**FIGURE 1:**
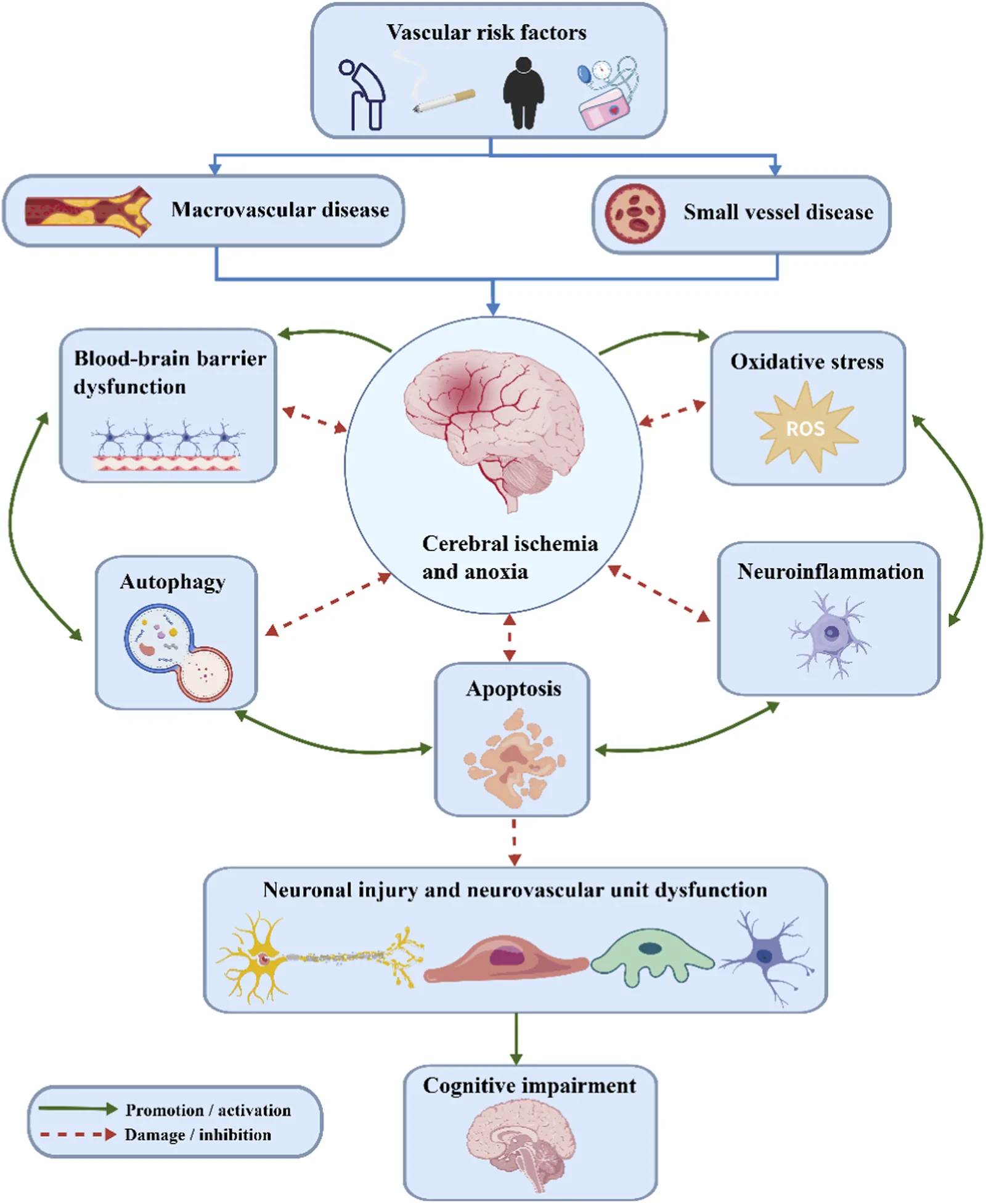
Pathological mechanism of vascular dementia, created with BioGDP.com (Jiang et al., 2025). Vascular risk factors (including aging, smoking, obesity, hypertension, etc.) can lead to macrovascular disease and cerebral small vessel disease. Vascular lesions further cause cerebral ischemia and hypoxia, thereby inducing a series of pathological processes such as oxidative stress, neuroinflammation, apoptosis, autophagy disorders, and blood-brain barrier damage. These pathological mechanisms interact with each other and form a vicious cycle, eventually leading to neuronal damage and neurovascular unit dysfunction. As the function of neurons, endothelial cells, glial cells, and pericytes continues to be impaired, it eventually manifests as cognitive dysfunction and progresses to vascular dementia. The green arrow indicates promotion or activation, and the red dotted arrow indicates damage or inhibition.

## 4 Natural products in vascular dementia: neuroprotection and neural repair

The pathological course of VaD is dynamic and strongly dependent on disease duration. Persistent cerebral hypoperfusion can initiate interconnected changes involving oxidative stress, neuroinflammation, BBB dysfunction, and neuronal and white matter injury. These processes follow different temporal trajectories, however, and their duration and relative contribution depend on hypoperfusion severity and experimental context. In CCH models such as two-vessel occlusion (2-VO), BBB disruption can occur early, whereas glial responses, neuronal and white matter injury, and subsequent tissue remodeling show distinct temporal patterns (Sun et al., 2021). Given the marked temporal heterogeneity of these pathological processes, time points such as 3, 7, 14, and 28 days are better regarded as representative temporal windows for characterizing the evolution of pathology in experimental cerebral hypoperfusion, rather than as standardized acute, subacute, and chronic stages of VaD (Sun et al., 2023).

Within this dynamic context, natural products may influence both neuroprotection and neural repair. Limiting secondary injury can preserve vascular and cellular substrates needed for later recovery, while vascular remodeling, endogenous neurogenesis, and synaptic remodeling may contribute to restoration of neural function. These processes are shaped by disease state and local tissue conditions and may overlap rather than occur as a discrete transition from one therapeutic phase to another (Figures 2, 3).

**FIGURE 2:**
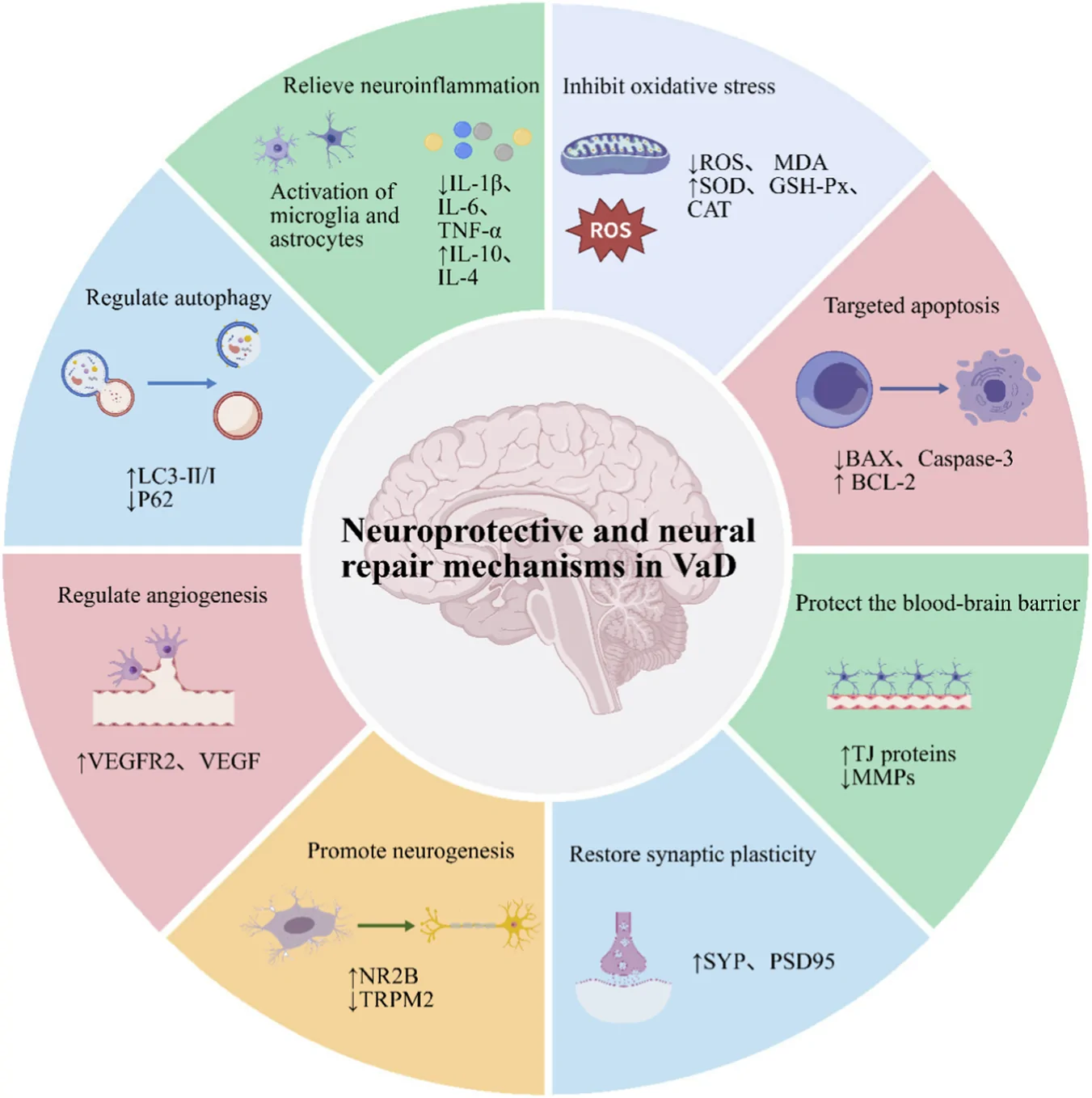
Therapeutic framework linking neuroprotection and neural repair in vascular dementia, created with BioGDP.com (Jiang et al., 2025). Vascular injury and cerebral hypoperfusion initiate interconnected pathological processes involving oxidative stress, neuroinflammation, neuronal injury, autophagy–lysosome dysregulation, and BBB dysfunction, which contribute to secondary tissue damage and neurological dysfunction. In parallel, vascular remodeling, neurogenesis, and synaptic plasticity represent endogenous adaptive responses that may contribute to tissue and functional recovery. Neuroprotective and neural repair processes are not strictly sequential but may overlap throughout disease progression. The framework summarizes major pathological mechanisms and therapeutic priorities relevant to vascular dementia and provides a conceptual basis for understanding how natural products may modulate multiple interconnected targets and pathways. (The “↑” means upregulated; the “↓” means downregulate).

**FIGURE 3:**
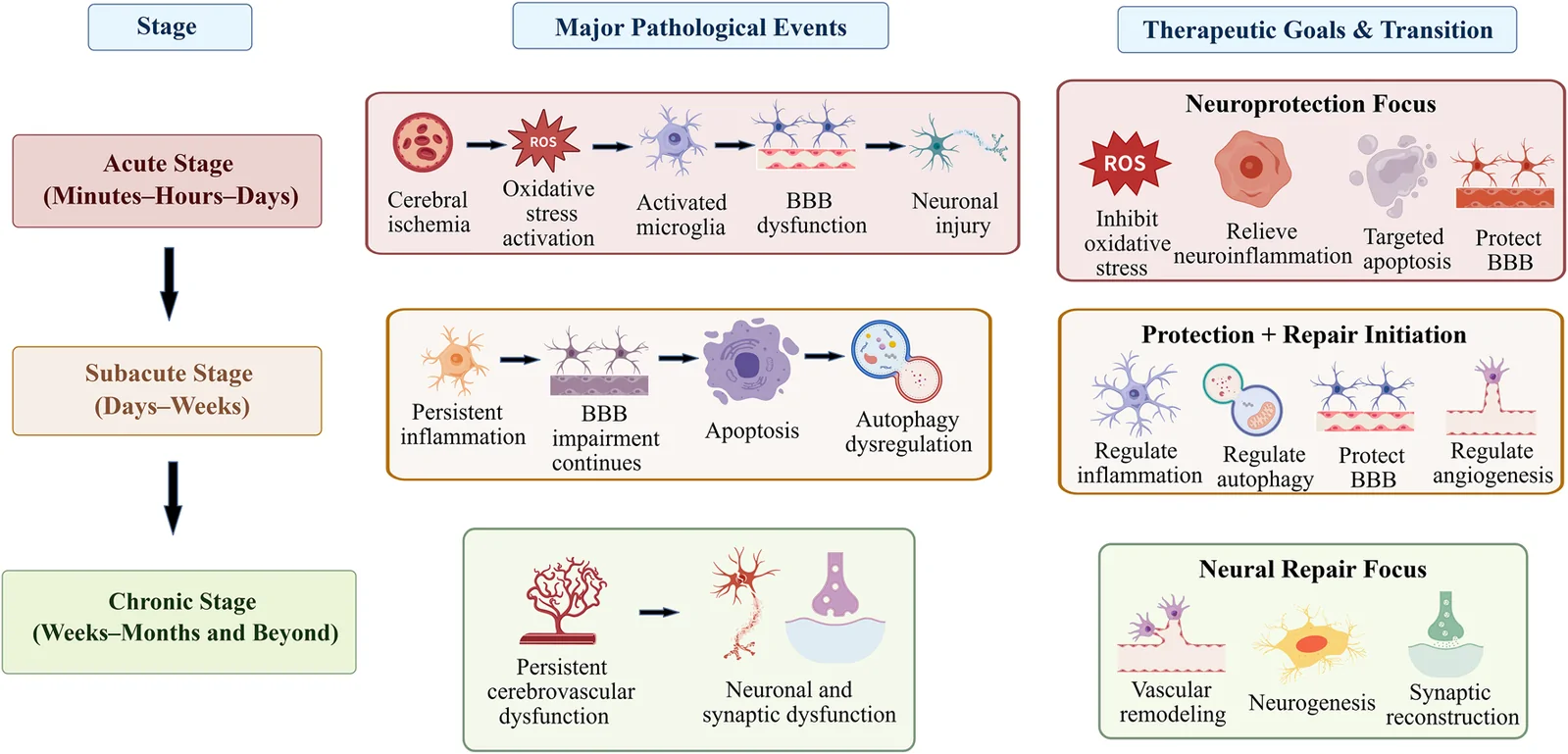
Temporal integration of pathological processes and therapeutic priorities in vascular dementia, created with BioGDP.com (Jiang et al., 2025). Vascular injury and cerebral hypoperfusion initiate a dynamic cascade involving metabolic stress, oxidative stress, neuroinflammation, BBB dysfunction, neuronal injury, and subsequent white matter and synaptic abnormalities. These processes are interdependent and may persist or evolve over different time courses, with their relative contribution influenced by the severity and duration of hypoperfusion and the characteristics of the experimental model. As tissue injury progresses, vascular remodeling, autophagy–lysosome homeostasis, endogenous neurogenesis, and synaptic plasticity may become increasingly relevant to tissue adaptation and repair. Accordingly, the therapeutic emphasis of natural products may extend from early neuroprotection aimed at limiting secondary injury toward increasing support for vascular and neural repair, while neuroprotective and reparative processes remain overlapping rather than strictly sequential. The temporal windows shown in the figure are conceptual representations derived mainly from experimental vascular injury and chronic cerebral hypoperfusion models and should not be interpreted as standardized clinical stages of VaD.

The following sections consider these effects according to the major pathological processes through which natural products may influence neuroprotection and neural repair, with emphasis on the relationship among molecular targets, tissue responses, and functional outcomes.

### 4.1 Neuroprotective strategies

Neuroprotection is an important therapeutic objective throughout the course of VaD, with the aim of limiting secondary brain injury and preserving neurovascular-unit function. Chronic cerebrovascular dysfunction can disturb redox homeostasis, inflammatory signaling, neuronal survival, autophagy–lysosome function, and BBB integrity. These processes are closely linked and together contribute to neuronal, white matter, and synaptic injury. Natural products may intervene at different points in this network, but the biological significance of a given target is best judged by whether its modulation is accompanied by protection of tissue structure and function.

#### 4.1.1 Inhibit oxidative stress

Prolonged cerebral hypoperfusion in VaD places metabolic and oxidative stress on brain tissue. Cardiovascular risk factors may further reduce endothelial nitric oxide availability while increasing reactive oxygen species (ROS) generation (Förstermann et al., 2017). The resulting redox imbalance can damage lipids, proteins, mitochondria, and cellular signaling systems. Natural products reported in VaD models appear to act at different levels of this response rather than through a single antioxidant mechanism.

One level involves pathways that sense redox stress and regulate endogenous defense. The Keap1/Nrf2/HO-1 axis is a central regulator of antioxidant responses. GJ-4 reduced white matter injury and improved cognitive performance in association with activation of this pathway (Pang et al., 2024), whereas fisetin combined Nrf2/HO-1 activation with suppression of the NF-κB/NLRP3 pathway *in vitro* (Cordaro et al., 2022). By contrast, puerarin acts on a redox-sensitive TRPM2/NMDAR pathway; in BCCAO rats, its inhibition reduced excessive ROS production (Zhu et al., 2021). These findings suggest that antioxidant effects may arise from distinct upstream interventions, ranging from enhancement of transcriptional defense to modulation of redox-sensitive ion-channel signaling.

Natural products may also strengthen the biochemical buffering capacity of the cell. SOD, catalase (CAT), and glutathione peroxidase (GSH-Px) form major components of this system (Casado et al., 2008; Chen et al., 2022). Ethanol extract of *Rubia yunnanensis* Diels reduced ROS and MDA while increasing SOD and GSH-Px activities (Cheng et al., 2025). Fo-Shou-San likewise improved glutathione redox homeostasis and modulated NRF2/HO-1–associated ferroptosis in an rUCCAO model (Wang J. et al., 2023). These studies indicate that restoration of antioxidant capacity may represent a common downstream effect even when the upstream targets differ.

At the downstream level, oxidative injury is commonly reflected by the accumulation of lipid peroxidation products such as MDA and 4-hydroxynonenal. Although these markers provide an indication of oxidative burden, they are not specific molecular targets. Accordingly, reductions in MDA or ROS provide more informative mechanistic evidence when accompanied by improvements in upstream signaling, mitochondrial function, ferroptosis-related processes, and neuronal or white matter outcomes.

Clinical evidence offers a limited but more direct link between redox biomarkers and treatment response. In patients with VaD, Dengzhan Shengmai Capsules combined with butylphthalide reduced serum lipid peroxides and MDA and increased SOD activity, alongside cognitive improvement (Ma and Sun, 2020). Because this was a combination intervention and the biomarkers were measured in peripheral blood, the findings support an association between treatment and systemic redox status rather than direct modulation of a cerebral antioxidant pathway.

#### 4.1.2 Relieve neuroinflammation

Neuroinflammation triggered by ischemic or hypoxic injury is an important component of VaD pathology (Belkhelfa et al., 2018). Activation of microglia and astrocytes is accompanied by increased production of cytokines, chemokines, reactive mediators, and inflammasomes (DiSabato et al., 2016; Tian et al., 2022). These signals can reinforce glial activation and sustain neuronal and white matter injury. Natural products therefore act on more than the final concentration of inflammatory mediators; their effects may occur at the level of immune-signal initiation, glial state, or inflammasome activation.

TLR4/MyD88/NF-κB signaling is a major point of convergence for innate immune activation. Baicalein suppresses this axis while activating Wnt/β-catenin signaling (Song J. et al., 2024; Xiao et al., 2023). Puerarin has likewise been linked to TLR4/MyD88/NF-κB signaling, with pharmacological TLR4 activation attenuating its protective effects (Tang et al., 2026). Resveratrol acts at a different upstream node by suppressing STING/TBK1/IRF3 signaling in CCH(Kang et al., 2022). Together, these findings place several natural products upstream of inflammatory amplification, but they do not establish that all downstream inflammatory changes are mediated by a single common target.

A second level involves inflammasome activation and inflammatory cell-death pathways. CCH can promote the activation of the NLRP3 and AIM2 inflammasomes, which are associated with microglial activation and increased production of the pro-inflammatory cytokines IL-1β and IL-18 (Matsuyama et al., 2020; Poh et al., 2021). In a 2-VO rat model, 20(S)-protopanaxadiol (PPD) was reported to reduce Iba-1 expression and shift microglial polarization toward an anti-inflammatory phenotype, thereby attenuating NLRP3 inflammasome-mediated neuroinflammation and reducing the neurotoxic effects associated with Aβ deposition and Tau hyperphosphorylation (Wang X. et al., 2023).

The functional state of glial cells may determine whether inflammation remains damaging or contributes to debris clearance and repair. In chronic white matter injury, broad suppression of glial activity may therefore be less desirable than limiting sustained pro-inflammatory signaling while preserving reparative functions. Quercetin promoted a shift toward an anti-inflammatory microglial phenotype and reduced demyelination (Tan et al., 2022), whereas QBT attenuated glial activation and neuroinflammation through the CXCL12/CXCR4 axis (Ma et al., 2024). This distinction is important because the therapeutic effect of a natural product may depend not only on reducing inflammation, but also on restoring a glial state compatible with tissue repair.

Clinical evidence is currently stronger for changes in global inflammatory or cognitive outcomes than for direct target engagement. In a randomized trial of Sailuotong, cognitive benefit was observed at 26 weeks, but the primary cognitive difference was no longer statistically significant at week 52 (Jia et al., 2018). Because microglial states and inflammasome activity were not directly assessed, the trial does not establish that these pathways mediated the clinical response. It nevertheless illustrates the difficulty of connecting experimental anti-inflammatory mechanisms with durable clinical benefit.

#### 4.1.3 Targeted apoptosis

Neuronal apoptosis is an important downstream consequence of persistent cerebrovascular dysfunction and is closely linked to oxidative stress, neuroinflammation, mitochondrial dysfunction, and ER stress in VaD. The intrinsic pathway is governed largely by Bcl-2 family proteins and mitochondrial integrity, leading to cytochrome c release and activation of caspase-9 and caspase-3. The extrinsic pathway is initiated by death receptors of the tumor necrosis factor receptor superfamily and converges on executioner caspases (Verbrugge et al., 2010). ER stress is better regarded as an upstream cellular stress state that can promote apoptosis when the unfolded protein response fails to restore proteostasis (Zhang et al., 2023). Natural products may therefore limit apoptosis either by acting directly on apoptotic machinery or by restoring the cellular conditions that favor neuronal survival.

At the mitochondrial level, several natural products have been associated with preservation of the Bcl-2/Bax balance and reduced caspase activation. In a 2-VO rat model, resveratrol decreased Bax and caspase-3 while increasing Bcl-2, accompanied by improved cognition and synaptic plasticity (Kasaei et al., 2025). Similarly, imperatorin reduced Bax and caspase-3 levels while increasing Bcl-2 expression in a 2-VO model, thereby attenuating hippocampal neuronal injury and improving synaptic structure (Huang Y. et al., 2021).

Natural products may also act upstream of the apoptotic machinery by restoring pro-survival signaling and mitochondrial homeostasis. Macelignan, a lignan isolated from *Myristica fragrans* Houtt., has shown neuroprotective effects through mTOR activation, with associated restoration of mitochondrial function and attenuation of oxidative stress and neuronal apoptosis (Qi et al., 2026). NBP has likewise been reported to reduce autophagy- and apoptosis-associated injury and improve cognition in a VaD model, although the contribution of the Nrf2/SIRT3 pathway has not been established causally (Gao et al., 2022). These studies illustrate that anti-apoptotic effects may arise secondarily from restoration of cellular homeostasis rather than from direct inhibition of caspases.

ER stress provides another route by which vascular and metabolic disturbances can converge on neuronal apoptosis. GJ-4 reduced PERK/eIF2α/ATF4/CHOP signaling and caspase-3 activation in 2-VO rats, accompanied by reduced neuronal injury (Yuan et al., 2023). Taohong Siwu Decoction produced a similar pattern of reduced ER-stress-related signaling and apoptosis in a 2-VO model (Fan et al., 2024). The convergence of a botanical extract and a herbal formulation on ER-stress-associated apoptosis supports the pathway as a potentially shared mechanism, although the active constituents differ and the contribution of ER stress requires direct mechanistic validation.

A small clinical study provides limited molecular support for this mechanism. In patients with VaD, Shibing Xingnao Granules combined with acupuncture increased serum Bcl-2 and reduced Bax and caspase-3, alongside improvements in cognition and function (Liu H. et al., 2023). The small sample, combined intervention, and peripheral rather than brain-derived measurements prevent these findings from being interpreted as direct evidence of reduced neuronal apoptosis. They are better considered preliminary pharmacodynamic evidence.

#### 4.1.4 Regulate autophagy

Autophagy is a major intracellular quality-control mechanism for removing damaged proteins and organelles. In CCH-associated VaD, autophagic activity changes over time and can be either protective or detrimental depending on the duration and severity of ischemic stress. Moderate activation may support adaptation early after injury, whereas excessive or poorly resolved autophagy may become harmful later; persistent suppression of microglial autophagy may also sustain inflammation (Cai et al., 2025; Wang et al., 2021; Wu et al., 2025; Xu et al., 2023). The therapeutic question is therefore not simply whether to increase or decrease autophagy, but whether the balance between autophagic flux and lysosomal degradation is restored in the relevant cell type.

At the neuronal and synaptic levels, β-asarone and gastrodin have been reported to attenuate excessive or dysregulated autophagy. β-asarone reduced Beclin-1 and the LC3-II/LC3-I ratio while increasing p62, with associated recovery of mitochondrial and synaptic function and improved cognition through cAMP/PKA/CREB signaling (Ning et al., 2024). Gastrodin produced a similar reduction in Beclin-1 and LC3-II/LC3-I with increased p62, associated with PI3K/AKT activation (Chen et al., 2023). Their convergence suggests that suppressing excessive autophagic activity may protect neurons under sustained hypoperfusion, but changes in static autophagy markers do not by themselves demonstrate restoration of autophagic flux.

A different pattern is evident in white matter. PF11, an ocotillol-type saponin extracted from Panax quinquefolius, improved CCH-induced white matter injury by enhancing mTOR-mediated autophagy and lysosomal function in oligodendrocytes and reducing lysosomal defects (Wang et al., 2024). Thus, β-asarone and gastrodin appear to act mainly by restraining excessive neuronal autophagy, whereas PF11 acts to restore defective autophagy–lysosome processing in oligodendrocytes. This contrast underscores the importance of cell type and autophagic context when interpreting therapeutic effects.

Clinical studies have not yet directly measured autophagic flux or lysosomal function in patients with VaD. Consequently, the proposed benefit of natural products on autophagy–lysosome homeostasis remains a predominantly preclinical finding, and whether these cellular effects contribute to clinical recovery is unresolved.

#### 4.1.5 Protect the blood-brain barrier

The BBB is a key component of the neurovascular unit and an important interface between cerebrovascular dysfunction and brain parenchymal injury. Cerebral ischemia and hypoxia can increase BBB permeability, while oxidative stress and inflammation may further aggravate barrier disruption (Janyou et al., 2017). Pericyte loss is also implicated in BBB breakdown. In CCH, BBB dysfunction can emerge early and precede prominent neuroinflammatory responses and white matter lesions (Sun et al., 2021). Maintaining barrier integrity may therefore interrupt the progression from vascular dysfunction to secondary tissue injury.

Natural products may preserve BBB integrity by acting on multiple components of the vascular barrier and its surrounding microenvironment. At the structural level, they may support the stability of vascular endothelial cells and pericytes. *Artemisia annua* Linné has been reported to preserve BBB integrity by increasing the expression of the endothelial adhesion molecule PECAM-1, the pericyte marker PDGFRβ, and the tight junction protein occludin, thereby maintaining neurovascular unit integrity (Kim et al., 2023). Natural products may also modulate endothelial stress responses and metabolism-related signaling. For example, melatonin has been shown to attenuate BBB disruption through activation of the SIRT1/PGC-1α/PPARγ signaling pathway, thereby exerting neuroprotective effects (Thangwong et al., 2023).

The consequences of BBB dysfunction extend beyond alterations in vascular permeability. Once endothelial and pericyte dysfunction is established, it may further promote neuroinflammation, oligodendrocyte injury, demyelination, and axonal dysfunction. *In vitro* studies have shown that Epimedium extract can attenuate demyelination and axonal injury by improving BBB dysfunction, an effect associated with inhibition of TNF signaling (Xie et al., 2022).

Clinical evidence currently provides mainly indirect support for vascular protection. In a randomized controlled trial of WeiNaoKang in patients with VaD, improvements in cognitive function were accompanied by changes in cerebral blood flow perfusion in selected brain regions (Liu et al., 2022). However, improved cerebral perfusion does not by itself demonstrate restoration of BBB permeability or neurovascular unit integrity. Thus, the protective effects of natural products on the BBB and neurovascular unit remain supported predominantly by preclinical evidence, and their clinical relevance requires further validation using integrated assessments of BBB permeability, cerebral perfusion, and vascular unit-associated biomarkers.

### 4.2 Neural repair strategies

Neural repair complements neuroprotection by supporting structural and functional recovery of injured brain tissue. In VaD, recovery may involve improved regulate angiogenesis, promote neurogenesis, and restore synaptic plasticity. These processes influence one another and remain constrained by the extent of residual tissue injury and the local microenvironment. Evidence for repair should therefore extend beyond increases in individual markers and include changes in tissue structure, cerebral perfusion, and functional performance.

#### 4.2.1 Regulate angiogenesis

Angiogenesis is a regulated process in which pro-angiogenic and anti-angiogenic signals coordinate basement membrane degradation, endothelial cell (EC) activation, proliferation, and migration, ultimately leading to the formation of mature vascular networks from pre-existing capillaries (Liu Z. L. et al., 2023). Disruption of angiogenic homeostasis represents an important component of VaD pathology. Insufficient angiogenesis may severely constrain endogenous tissue repair and limit the restoration of cerebral perfusion, whereas excessive or dysregulated angiogenesis may promote pathological neovascularization, vascular leakage, and tissue dysfunction (Rust et al., 2019). Therefore, vascular restoration in VaD should focus on the formation of functional vascular networks and adaptive vascular remodeling rather than simply enhancing pro-angiogenic signaling.

Natural products may target different components of the angiogenic cascade. At the level of pro-angiogenic signaling, dihydromyricetin (DMY) has been reported to increase cerebral blood flow, attenuate neuronal injury, promote angiogenesis, and improve cognitive impairment, effects associated with activation of the VEGFR2 pathway (Zhou et al., 2025). In a BCCAO rat model, baicalein was also shown to improve angiogenesis and suppress neuroinflammation by upregulating SIRT1 expression and downregulating Notch1-related signaling (Li et al., 2025). These findings suggest that angiogenesis does not occur in isolation but may evolve in parallel with the inflammatory microenvironment and neuronal survival.

Some naturally derived compounds may further coordinate angiogenesis with other reparative processes. In 2-VO rats, NBP activated the Shh/Ptch1 signaling pathway, accompanied by increased expression of VEGF, FGF-1, and Ang, enhanced angiogenesis, and reduced autophagy. Silencing Shh partially abolished NBP-induced angiogenesis and functional improvement, supporting a role for Shh/Ptch1 signaling in NBP-mediated vascular remodeling and neurological recovery (Niu et al., 2021).

From a clinical perspective, evidence concerning cerebral perfusion is currently more informative than direct evidence of angiogenesis. NBP has been evaluated in randomized controlled trials involving patients with vascular cognitive impairment; however, these studies have primarily focused on cognitive and functional outcomes and have not directly demonstrated modulation of angiogenic pathways such as VEGFR2, HIF-1α/VEGF, or Shh/Ptch1 (Jia et al., 2016). Thus, the angiogenic effects identified in animal studies should currently be regarded as potential mechanisms underlying neural repair. Whether these mechanisms translate into sustained improvements in cerebral perfusion and neurological function in patients remains to be established.

#### 4.2.2 Promote neurogenesis

Neurogenesis refers to the regenerative process by which neural stem/progenitor cells proliferate, differentiate, and mature to generate new neurons (Emsley et al., 2005). In adult mammals, neurogenesis has been most consistently associated with the hippocampal dentate gyrus, particularly the subgranular zone, and may contribute to learning and memory processes (Spalding et al., 2013). CCH can promote senescence and depletion of hippocampal neural stem cells, while a persistent inflammatory and metabolically unfavorable microenvironment may further restrict endogenous neurogenesis and contribute to cognitive dysfunction. Thus, enhancing the survival and regenerative capacity of neural stem/progenitor cells may represent an important component of neural repair in VaD.

Natural products may modulate different stages of the neurogenic process. For example, floralozone is a colorless liquid originally identified in *Lagotis* Gaertn. Pharmacological studies have shown that floralozone improves the morphology and structural integrity of hippocampal neurons, reduces neuronal apoptosis, promotes neurogenesis, and ameliorates cognitive impairment. These effects have been associated with activation of AMPKα2, which increases miR-7a-5p expression, thereby suppressing TRPM2 and enhancing N-methyl-D-aspartate receptor subunit 2B (NR2B) expression (Song Y. T. et al., 2024).

Traditional Chinese medicinal preparations have also been reported to promote neurogenesis after vascular injury. Recent evidence from a bilateral common carotid artery stenosis (BCAS) mouse model showed that Xinshubao tablets promoted neurogenesis and alleviated cognitive deficits and neuronal injury, potentially through inhibition of NF-κB signaling (Xiao et al., 2024). Similarly, QNYZD has been reported to promote neurogenesis, an effect associated with suppression of JAK2/STAT3 activation (Wu et al., 2015).

Clinical evidence supporting enhanced neurogenesis remains limited. Existing clinical studies of vascular cognitive impairment or VaD have primarily evaluated cognitive function, activities of daily living, or cerebral perfusion, rather than directly assessing hippocampal neurogenesis in patients. Accordingly, improvements in cognitive performance may represent a functional consequence of neural network recovery, but they cannot by themselves establish that hippocampal neurogenesis has been enhanced. Direct clinical validation of neurogenic mechanisms will require biomarkers or imaging approaches capable of linking changes in neurogenesis to structural and functional outcomes.

#### 4.2.3 Restore synaptic plasticity

Transient or sustained cerebral ischemia and hypoxia can result in persistent deficits in synaptic transmission, potentially related to impaired phosphorylation of presynaptic proteins (Bolay et al., 2002). Alterations in the levels of synaptic proteins, including synaptosomal-associated protein 25, bassoo, and excitatory amino acid transporter 2, suggest the engagement of compensatory mechanisms during the progressive synaptic deterioration associated with VaD. Postsynaptic density protein 95 (PSD-95), in particular, plays a complex and critical role in the pathological processes of cerebral ischemia and dementia, and its dysfunction may exacerbate both neuronal injury and synaptic failure (Gallart-Palau et al., 2015; Sinclair et al., 2015). However, synaptic injury in VaD is not simply characterized by a reduction in individual synaptic proteins but involves multiple interconnected processes, including maintenance of synaptic architecture, neurotransmitter–receptor signaling, and activity-dependent synaptic plasticity.

Several natural products may modulate synaptic plasticity by preserving synaptic structure or restoring neurotransmitter–receptor signaling. *In vivo* studies have shown that Dendrobium polysaccharides improve cognitive performance by attenuating ferroptosis and restoring synaptic plasticity. These effects were accompanied by increased expression of GSH, xCT, GPx4, and PSD-95, as well as increases in the number of synaptic vesicles, the length of the presynaptic active zone, and the thickness of the postsynaptic density (Ming et al., 2023). Similarly, in a 2-VO rat model, naringenin enhanced cognitive performance by increasing the hippocampal expression of synaptophysin (SYP), PSD-95, N-methyl-D-aspartate receptor 1, and NR2B, thereby promoting N-methyl-D-aspartate receptor (NMDAR)-mediated synaptic plasticity (Zhang et al., 2022).

Beyond synaptic proteins themselves, neurotrophic and activity-dependent signaling pathways may also contribute to synaptic remodeling. Tetrahydrocurcumin has been reported to attenuate oxidative stress and neuronal injury through activation of the Nrf2 pathway and to improve synaptic protein expression and plasticity through the Wnt/β-catenin/BDNF/TrkB/CREB signaling cascade (Jearjaroen et al., 2024). Synaptic remodeling also depends on intact axonal transport and adequate local energy supply. Recent evidence suggests that *Strychni Semen* and its active constituent strychnine, but not rucine, promote axonal regeneration and alleviate white matter injury by enhancing axonal mitochondrial motility, highlighting mitochondrial transport and local energy provision within axons as potentially important components of restoring damaged neural connectivity (Zhang Y. et al., 2026).

Direct clinical evidence demonstrating restoration of synaptic plasticity by natural products in patients with VaD remains lacking. Cognitive benefits observed in clinical studies may indicate improvements in neural network function, but they cannot by themselves establish recovery of hippocampal synaptic architecture or activation of plasticity-associated pathways such as NMDAR and BDNF/TrkB/CREB signaling.

In order to construct a rigorous and comprehensive review, Table 1 summarizes recent studies of isolated natural compounds, botanical extracts, and herbal formulations investigated in vascular dementia, with a focus on their sources, experimental models, dosing regimens, and reported molecular targets or signaling pathways.

**TABLE 1:** Preclinical evidence for natural products in vascular dementia.

Category	Source	Natural products	*In vitro*	*In vivo*	Dosage and duration	Primary mechanism	Key targets/pathways	Reference
Isolated natural compounds	Polyphenols	Rosmarinic acid	OGD-exposed microglial models	BCAS mice model	50, 100 mg/kg/d i.p ×56 days	Inhibit oxidative stress and relieve neuroinflammation	Regulate Nrf2/HO-1 and NF-κB–NLRP3 signaling	(Jiang et al., 2026)
Isolated natural compounds	Polyphenols	Farrerol	N/A	BCCAO rat model	20, 40 mg/kg/d i.p ×28 days	Relieve neuroinflammation	Inhibit the MAPK-NF-κB signaling pathway	(Zhang et al., 2025)
Isolated natural compounds	Polyphenols	Puerarin	N/A	rUCCAO mice model	200, 400 mg/kg/d i.p ×10 days	Relieve neuroinflammation and promote myelin regeneration	Enhance the IL-10/STAT3/CD36 signaling pathway	(Luo et al., 2025)
Isolated natural compounds	Saponin	Ginsenoside Rg1	N/A	2-VO rat model	10 mg/kg/d i.p ×35 days	Restore amino acid homeostasis and neurotransmitter equilibrium	Activate Adcy1/Kdr–mediated cholinergic synapse and PI3K–AKT signaling pathway	(Wang et al., 2025)
Isolated natural compounds	Saponin	Astragaloside IV	N/A	rUCCAO mice model	20, 40 mg/kg/d i.p ×28 days	Inhibit oxidative stress and improve white matter damage	Activate the SIRT1/Nrf2 signaling pathway	(Ma et al., 2023)
Isolated natural compounds	Phenylpropanoids	Byakangelicin	N/A	2-VO rat model	10, 30 mg/kg/d i.p ×28 days	Relieve neuroinflammation and inhibit apoptosis	Regulate the KDR–NOS3 signaling axis and VEGF/Akt/eNOS pathway	(Wang et al., 2026)
Isolated natural compounds	Anthraquinone	Alizarin	N/A	2-VO rat model	2.5, 8, 25 mg/kg/d i.g ×28 days	Inhibit oxidative stress	Increase the ubiquitination and degradation levels of TRPM2	(Pan et al., 2024)
Isolated natural compounds	phenolic glycoside	Gastrodin	N/A	BCCAO rat model	25, 50 mg/kg i.g ×56 days	Alleviate mitochondrial dysfunction	Regulate the SIRT3/TFAM acetylation/OXPHOS/mitochondrial dynamic balance	(Chen et al., 2024)
Isolated natural compounds	Phthalide	DL-3-n-butylphthalide	N/A	BCAS mice model	80 mg/kg i.g ×28 days	Promote synaptic plasticity and decrease BBB leakage	Activate the AKT/ERK signaling pathway and inhibit the HIF-1α/MMP signaling pathway	(Che et al., 2023)
Isolated natural compounds	Phthalide	A phthalein component	OGD/R-induced HT22 cell model	2-VO rat model	13.5, 27, 54 mg/kg i.g ×28 days	Inhibit ferroptosis and pyroptosis of neurons	Activate the Nrf2/xCT/GPX4 pathway and inhibit the NLRP3/Caspase-1/GSDMD pathway	(Feng et al., 2024)
Botanical extracts	Extract	*Ginkgo biloba* extract	OGD/R-induced SH-SY5Y cell model	Recurrently clipping the common carotid arteries and intraperitoneal injections of sodium nitroprusside solution	50 mg/kg i.g ×30 days	Reduce apoptosis and autophagy	Inhibit AMPK-mTOR	(Yin et al., 2024)
Botanical extracts	Extract	Methanol extract of *Ligusticum chuanxiong* Hort. Rhizome	N/A	BCAS mice model	5, 50, 500 mg/kg i.g ×6 weeks (5 times/week)	Relieve neuroinflammation	Inhibit activation of astrocytes and microglia	(Lim et al., 2024)
Botanical extracts	Extract	*Gastrodia elata* ethyl acetate extract	OGD-induced HT22 cell model	2-VO rat model	34,102 mg/kg i.g ×28 days	Relieve neuroinflammation	Regulate the AMPK/SIRT1/FoxO1/NF-κB signaling pathway	(Tao et al., 2026)
Herbal formulations	Formula	Shibing Xingnao formula	OGD/R-induced HT22 cell model	2-VO rat model	3, 6, 12 g/kg i.g ×28 days	Improve synaptic plasticity	Activate the cAMP/PKA/CREB/BDNF signaling pathway	(Gao et al., 2025)
Herbal formulations	Decoction	Ditan Decoction	N/A	2-VO rat model	2.14, 4.28, 8.56 g/kg i.g ×28 days	Inhibit ferroptosis	Activate the HIF1α pathway	(Gu et al., 2025)
Herbal formulations	Decoction	Zishen Huoxue Decoction	N/A	2-VO rat model	8.9, 17.8, 35.6 g/kg i.g ×28 days	Inhibit apoptosis	Upregulate the PINK1/Parkin signaling	(Zhao et al., 2024)
Herbal formulations	Decoction	Guipi Tang	N/A	BCCAO rat model	3.1, 6.2, 12.4 g/kg i.g ×28 days	Regulate gut microbiota	Inhibit the Vax1-AK8 pathway	(Ren et al., 2025)
Herbal formulations	Decoction	Modified Erchen decoction	N/A	2-VO rat model	2.5, 5, 10 g/kg i.g ×28 days	Activate blood and eliminate phlegm	Inhibit the JAK2/STAT3 and JNK/BAX signaling pathways	(Gao et al., 2023)
Herbal formulations	Decoction	Tong-Qiao-Huo-Xue Decoction	OGD/R-induced HT22 cell model	2-VO rat model	3, 6, 12 mg/kg i.g ×28 days	Improve synaptic plasticity	Activate the cAMP/PKA/CREB pathway	(Li et al., 2024)
Herbal formulations	San	Danggui-Shaoyao San	Establishment of hypoxia PC12 cell model by CoCl_2_	2-VO rat model	5, 10 g/kg i.g ×28 days	Improve synaptic plasticity	Enhance the HIF-1α/EPO axis	(Ningning et al., 2024)
Herbal formulations	Capsule	Qizhi Tongluo capsule	N/A	2-VO rat model	0.3, 0.6,1.2 g/kg i.g ×28 days	Inhibit oxidative stress, relieve neuroinflammation, anti-apoptosis	Regulate glycerophospholipid, amino acid, and fatty acid, metabolism and metabolic pathways	(Sun et al., 2026)
Herbal formulations	Wan	YangXue QingNao Wan	Erastin-induced HT22 cell model	2-VO rat model	250, 500, 1000 mg/kg i.g ×36 days	Inhibit ferroptosis	Inhibit the Dipeptidyl Peptidase 4 expression	(Zhang Y.-J. et al., 2026)
Herbal formulations	Granules	Tongqiao Huashuan granules	OGD/R-induced SH-SY5Y cell model	2-VO rat model	1.35, 2.7,5.4 g/kg i.g ×28 days	Inhibit oxidative stress and improve intracellular calcium overload	Activate the PLCγ1/IP3R pathway	(Huang et al., 2026)

## 5 Discussion

Current evidence suggests that neuroprotection and neural repair are closely linked in VaD rather than representing two independent therapeutic phases. Cerebrovascular dysfunction, BBB impairment, oxidative stress, and neuroinflammation can interact and progressively affect neuronal survival, white matter integrity, and synaptic function. The extent of this initial and secondary injury may, in turn, influence the capacity for vascular remodeling and subsequent recovery of neural networks. Neuroprotective and neural repair processes should therefore be considered overlapping components of disease modification rather than interventions confined to predefined stages. For natural products, this perspective is particularly relevant because changes in a single signaling pathway may not adequately reflect their overall biological effects, which are better interpreted in relation to tissue protection and functional outcomes (Ghorani-Azam et al., 2018).

The natural products examined in this review comprise isolated natural compounds, botanical extracts, and herbal formulations, and these categories differ in both pharmacological interpretation and translational requirements. Isolated compounds generally have defined chemical structures and identifiable molecular targets, allowing more direct assessment of target specificity and dose–response relationships (Wąsik and Antkiewicz-Michaluk, 2017). Botanical extracts and herbal formulations are chemically more complex, and their effects may reflect the combined actions of several constituents. Their pharmacological interpretation is consequently affected by differences in absorption, metabolism, constituent interactions, and batch composition. Such factors can complicate both mechanistic attribution and product standardization (Madanahalli Ramesh et al., 2026). For isolated compounds, target validation and quantitative pharmacological characterization are therefore particularly important, whereas studies of extracts and herbal formulations should additionally establish chemical fingerprints, quality markers, active constituents, and batch consistency.

The mechanistic evidence is strongest when changes in a candidate pathway are accompanied by evidence linking target modulation to tissue effects and functional improvement. Dihydromyricetin provides a useful example. VEGFR2 emerged as a candidate target from network pharmacology and molecular docking, and its interaction with dihydromyricetin was subsequently examined using molecular dynamics simulations and surface plasmon resonance. In BCAS mice and hypoxic/hypoglycemic endothelial cells, DMY promoted angiogenesis, increased cerebral blood flow, and improved cognitive performance, whereas VEGFR2 inhibition weakened these effects (Zhou et al., 2025). This combination of target-level and phenotypic evidence provides substantially stronger support than studies in which pathway activation is inferred only from changes in protein expression. Macelignan provides another example. Transcriptomic analysis and molecular modeling implicated mTOR in its activity, while subsequent experiments linked mTOR activation to mitochondrial homeostasis, antioxidant responses, mitophagy, and apoptosis. Importantly, inhibition of mTORC1 with rapamycin abolished the protective effects of macelignan, providing pharmacological evidence that mTOR is not merely associated with, but contributes functionally to, its neuroprotective action (Qi et al., 2026). Even so, both examples remain based on preclinical models.

Although preclinical mechanistic studies of natural products continue to accumulate, clinical validation of these mechanisms in humans remains limited, highlighting a translational gap between preclinical findings and clinical benefit (Table 2). A recent systematic review of randomized controlled trials identified 16 trials involving 5,668 participants; only a small proportion evaluated natural or herbal interventions. EGb 761® was studied in three trials and showed a signal for improvement in SKT scores, but substantial heterogeneity was observed (I^2^ = 92.8%) and the certainty of evidence was rated very low. Tianzhi granule was examined in only one trial, leaving its clinical efficacy uncertain (Cheng et al., 2026). These findings do not establish consistent patient-important benefits of natural products in VaD. More importantly, clinical improvements in cognition or other functional measures cannot be assumed to reflect engagement of the molecular targets identified in experimental studies.

**TABLE 2:** Clinical evidence for natural products in vascular dementia.

Natural product	Sample size	Dosage and duration	Clinical outcomes	Safety/key limitations	Reference
Cannabidiol	30	300 mg/day ×4 weeks	CBD significantly reduced NPI and BPRS scores *versus* placebo; no significant differences in cognitive or functional outcomes	Adverse events were mild and similar between groups; small sample, short duration, no demonstrated cognitive/functional benefit	(Pessoa et al., 2026)
Tianzhi granule	543	5 g, three times daily ×24 weeks	Significantly improved VADAS-cog and CIBIC-plus *versus* placebo; no significant difference from donepezil	Symptomatic benefit demonstrated over 24 weeks; long-term efficacy and disease-modifying effects remain uncertain	(Shi et al., 2020)
Shenmayizhi formula + Ginkgo extract	196	Ginkgo extract tablets 1 tablet, orally, three times daily; SMYZF granules 4.8 g, orally, twice daily ×12 weeks	Greater improvement in MMSE, NIHSS and CM-SS than Ginkgo extract alone, with favorable changes in several serum biomarkers	No placebo-only comparator; adjunctive design, 12-week duration, 24 participants dropped out	(Zhang et al., 2020)
*Ginkgo biloba* extract EGb 761®	71	240 mg/day ×24 weeks	VaD-specific efficacy not independently established in the overall cohort	VaD subgroup was relatively small; long-term disease-modifying efficacy not established	(Ihl et al., 2012)

The gap between experimental efficacy and clinical applicability is also influenced by pharmacokinetic and formulation-related factors. For isolated compounds, dose, systemic concentration, tissue distribution, and pharmacodynamic response need to be considered together. For botanical extracts and herbal formulations, variation in chemical composition and constituent disposition introduces additional uncertainty into the relationship between treatment and biological response. This issue is particularly relevant in VaD because patients commonly receive multiple vascular medications, including antihypertensive, antiplatelet, anticoagulant, and antidiabetic agents, raising the possibility of pharmacokinetic or pharmacodynamic interactions. BBB permeability and potential neurotoxicity are also relevant when natural products are developed for CNS indications (Kato et al., 2025). These considerations suggest that candidate evaluation should extend beyond *in vitro* activity and changes in endpoint proteins toward quantitative dose–response analysis and pharmacodynamic measures that can be assessed in clinical settings.

The experimental models used to study VaD represent another source of uncertainty. Both 2-VO and BCAS reproduce important aspects of chronic cerebral hypoperfusion, but the extent and temporal profile of hypoperfusion and the resulting white matter and behavioral phenotypes differ from those observed across the heterogeneous clinical spectrum of VCID. Recent work on the BCAS model further illustrates that white matter injury evolves in a spatially and temporally heterogeneous manner, emphasizing the importance of considering lesion distribution and disease duration when interpreting experimental outcomes (Kane et al., 2026; Mun et al., 2026). Thus, the choice of model may influence both the apparent efficacy of an intervention and the mechanism assigned to that effect. Future studies would benefit from matching models more closely to the pathological process under investigation and, where possible, confirming findings across complementary experimental settings. Clinical trials should likewise account for vascular etiology, cognitive severity, and coexisting neurodegenerative pathology when evaluating natural-product interventions. Integrating pathological phenotype, treatment response, mechanism-related biomarkers, and clinical outcomes should provide a more reliable basis for determining whether the multitarget properties of natural products offer a genuine therapeutic advantage in VaD.

## 6 Conclusion

VaD is a dynamic pathological process driven by persistent cerebrovascular dysfunction and shaped by complex interactions among vascular, immune, metabolic, and neural network alterations. Accumulating evidence indicates that isolated natural compounds, botanical extracts, and herbal formulations can target multiple pathological processes through mechanisms related to neuroprotection and neural repair, with potential benefits for brain tissue integrity and cognitive function in experimental models of VaD. However, the current evidence remains predominantly preclinical, and substantial differences exist in the strength of mechanistic and clinical evidence across different classes of natural products. Future clinical studies should integrate pharmacokinetic–pharmacodynamic profiles, target engagement, and mechanism-related biomarkers to better define the therapeutic value and clinical applicability of natural products in VaD.

## Glossary

AIM2: absent in melanoma 2
BBB: blood-brain barrier
BCL-2: B-cell lymphoma-2
BAX: BCL-2-associated X protein
BCCAO: bilateral common carotid artery occlusion
BCAS: bilateral common carotid artery stenosis
Caspase-3: cysteinyl aspartate-specific proteinase-3
CAT: catalase
CREB: cAMP response element binding protein
CCH: chronic cerebral hypoperfusion
CCL: chemokine (C-C motif) ligand
GPX4: glutathione peroxidase 4
GSH-Px: Glutathione peroxidase
IL-1β: interleukin-1β; IL-6, interleukin-6
TNF-α: tumor necrosis factor-α
IL-10: interleukin-10
IL-4: interleukin-4
LC3: microtubule-associated protein 1 light chain 3
MDA: malondialdehyde
MMPs: matrix metallopeptidases
NLRP3: NLR-pyrin domain containing 3
NMDAR: N-methyl-D-aspartate receptor
NR2B: N-methyl-D-aspartate receptor subunit 2B
NOX: nicotinamide adenine dinucleotide phosphate oxidase
OGD/R: oxygen-glucose deprivation/reperfusion
PSD95: postsynaptic density protein 95
P62: sequestosome-1
ROS: reactive oxygen species
SOD: superoxide dismutase
SYP: synaptophysin
TJ proteins: tight junction proteins
TRPM2: transient receptor potential melastatin 2
VaD: vascular dementia
VCID: vascular cognitive impairment and dementia
VEGF: vascular endothelial growth factor
VEGFR2: vascular endothelial growth factor receptor 2
2-VO: two-vessel occlusion
